# Emotional Contagion and Social Support in Pigs with the Negative Stimulus

**DOI:** 10.3390/ani13203160

**Published:** 2023-10-10

**Authors:** Ye Zhang, Jiaqi Yu, Yu Zhang, Yaqian Zhang, Fang Sun, Yuhan Yao, Ziyu Bai, Hanqing Sun, Qian Zhao, Xiang Li

**Affiliations:** 1 College of Animal Science and Technology, Northeast Agricultural University, Changjiang Road No. 600, Harbin 150030, China; zhangyeneau@163.com (Y.Z.); 18845721609@163.com (J.Y.); 15331802505@163.com (Y.Z.); yinyuxingzhou@163.com (Y.Z.); sunfang276@163.com (F.S.); yyh021023@126.com (Y.Y.); bzy050916@163.com (Z.B.); sunhq08@126.com (H.S.); zhaoqian@neau.edu.cn (Q.Z.); 2 Key Laboratory of Swine Facilities, Ministry of Agriculture, Northeast Agricultural University, Changjiang Road No. 600, Harbin 150030, China

**Keywords:** empathy, emotional contagion, negative stimulus, pig, social support

## Abstract

**Simple Summary:**

Emotional contagion and social support are common phenomena in animals. The study evaluated whether pigs that did not observe the stimulus process could also perceive the negative emotions of their companions and provide social support. Here, the research compared differences in behavioral responses between treated individuals and naive companions by measuring the frequency/duration and latency of behaviors occurring in pigs under different test conditions to determine whether there were behavioral responses of the companions with emotional contagion to support the treated individuals. Whether or not the companions were aware of the source of the negative emotions of the treated pigs, they were able to respond to it in an appropriate emotional way and provide social support to the treated individual.

**Abstract:**

This study expects to confirm the existence of emotional transmission in pigs from multiple perspectives and to provide theoretical references for improving animal welfare in livestock farming. A group that could directly observe (DO) and a group that could not directly observe (NO) were created based on whether or not their peers observed the treatment process, as the treated pig (TP) was treated with electrical shock and the companion pig (CP) either witnessed the treatment inflicted upon TP or not, and a third group was a control group, in which neither pig was stimulated. The behavioral responses of both the TPs and CPs were recorded to evaluate the emotional reaction. The results found that in both the DO and NO groups, the frequency of TP freezing was significantly higher than that of CP, and CP was significantly higher than that of the control group. Interestingly, although the social interaction responses of the CPs were not similar in the DO and NO groups, there were no significant differences between the behaviors of TPs in the DO and NO groups, except for nose–nose contact and a single approach to the partition, which allowed us to conclude that, whether or not the pigs directly observed the negative treatment, they were able to respond accordingly to fear and provide similar social support to their companions who were treated negatively.

## 1. Introduction

Empathy, the capacity to feel and understand another individual’s feelings in relation to oneself [1,2], includes cognitive empathy and emotional empathy. Cognitive empathy is the ability to understand the emotions of others, even when their emotional state is different from one’s own [3,4], whereas emotional empathy, also known as emotional contagion, is the most basic form of empathy [1,5]. The phenomenon of empathy is prevalent in nature and especially in livestock production, wherein animals do not only respond to direct exposure to environmentally harmful sources but also receive similar psychological and behavioral responses to biological information transmitted by specific individuals within the group [6,7]. The modern pig farming industry is characterized by intensification with high-density and standard procedures such as tail docking [8], castration [9], and pen exchange [10]. These traditional treatments lead to pain and stress to elicit negative emotions in some individual pigs, and then the empathy effect could spread negative emotions to others, which would affect pig welfare at the group level, thus endangering livestock production [6].

Pigs have a basic expression of empathy in social interactions, a phenomenon similar to emotional contagion [4,11,12,13]. Reimert et al. [11] demonstrated that pigs without any handling experience showed behaviors such as ears fixated backward and alert, standing after contact with demonstrator pigs that were negatively treated (social isolation); moreover, emotional contagion occurred between demonstrator pigs and naive penmates, and the naive penmates felt the negative emotions of the demonstrator pigs. When observer pigs saw their companions restrained, they were distressed and performed similar behaviors to demonstrator pigs such as freezing, escape attempts, and sharp sounds, demonstrating that negative emotional contagion occurred between demonstrator and observer pigs [4]. In the study by Reimert [12], after the handled pigs in the study received positive and negative treatments in their pens, their naive companions were influenced to show corresponding positive or negative behavioral performances, possibly due to emotional contagion. A study that trapped wild boars in cages through attractive food bait, and which recorded footage of their rescue by their peers, demonstrated rescue behavior among wild boars. Their rescue behavior was speculated to have been caused by the rescuer’s emotional state of congruence or understanding with the victim [13]. This also indicates that emotional contagion is a key step in promoting social support behaviors [14,15].

The ability of a social partner to reduce stress responses is commonly referred to as social buffering or social support [16,17]. Several lines of evidence suggest that social support begins with the generation of negative emotions in individuals who do not receive direct negative stimuli, that is, from the demonstrator inducing negative emotions in the observer [15,18]. For example, Reimert [11] found that urination and attempted escape behaviors were less frequent in training pigs with their naive penmates than without their presence and that training pigs could consider the presence of a companion as social support. In another study, under hypoxic conditions, treated pigs with a companion showed lower activity levels and fewer escape attempts compared to treated pigs without a companion, and treated pigs with a familiar companion spent more time in physical contact with their companion during hypoxia, possibly as the treated pigs sought for social support [17]. When demonstrator pigs were restrained, behaviors such as alert standing and escape attempts were decreased in demonstrator pigs with a companion compared to those without a companion, and the study found that social support had a significant effect on treatment pigs and demonstrated that pigs can benefit from social support [19]. Moreover, pigs are highly gregarious animals, which rely heavily on sensitive olfactory and tactile snouts for social nosing, a common contact behavior that aids in individual recognition and affiliation [20,21,22], including distinguishing social identity, creating dominance hierarchies [23], and identifying the emotional states of other pigs in aggressive encounters [24]. When demonstrator pigs were in distress due to restraint, the penmates increased their contact behaviors with them, such as nose contact, proximity, and head orientation, which could potentially provide social support for demonstration pigs [4]. Since the companion who can directly observe the stimulus process can provide social support through social interaction with the demonstrator pig, can the companion still provide social support to the demonstrator pig if it is not observed receiving the negative stimulus? The answer to this question is not yet known, which proves that emotional contagion occurs if the companion can still provide social support to the demonstrator without observing it undergoing the stimulus process.

Additionally, behavior in most studies is evaluated using frequency or duration, whereas behavioral latency is the time from some specified event that shows a sense of urgency to the events [25], which may be related to stronger impulses, and stronger emotions, but still needs to be verified [26]. In the Novelty Suppressed Feeding Test (NSFT), animals approaching and feeding in a novel open-field environment with an increased latency represent an anxious state [27,28,29]. Moreover, latency has been used to investigate aggressive behavior in sows, and it has been found that sows with a shorter latency to contact a model pig are more likely to show aggression when mixed [30]. Similarly, the response urgency of the pig to a negative stimulus could be judged based on the length of the behavioral latency to evaluate the willingness of the treated pig and its companion to contact after a negative stimulus.

Hereby, this study experimented with the condition of whether or not the companion could observe the treated pig receiving a punishment stimulus, and recorded the differences in behavioral responses between the treated and companion pigs, with the expectation of verifying that emotional contagion occurs between the treated pig and the companion, to explore the similarities and differences in social support responses given by peers to treated pigs under different conditions.

## 2. Materials and Methods

### 2.1. Animals and Management

This experiment was conducted from April to June at the experimental base in Shuangcheng District, Jiuyuan Village, Harbin, Heilongjiang Province. A total of 108 4-week-old pigs of similar weight (9.13 ± 0.13 kg) from different litters (1-week-old male depopulated, 4-week-old weaned) were housed in 9 m^2^ pens (3.60 × 2.50 × 1.00 m, 0.75 m^2^/pig), one for every 12 pigs, for a total of 9 pens. The pens were covered with rice husk of 0.50 m thickness as bedding without any supplement or replacement during the experiment. The pig house was naturally lit and the windows were opened for natural ventilation from 10:00 to 14:00 every day. The temperature was 20.00–25.00 °C (23.02 ± 1.92 °C) and the air humidity was kept at 65.00–75.00% (70.12 ± 2.96%). Water was available ad libitum, and the pigs were supplied with feed (energy of 3.30 MJ/kg, crude protein 17.00%, crude fiber 4.00%, lysine 1.30%) at 50 g/kg/pig/day, and were fed three times per day, at 7:00, 12:00, and 16:00, separately. All procedures were conducted by one person. The experimental animal feeding management was in accordance with the requirements of the Regulations on Experimental Animal Welfare and Ethical Management of Northeastern Agricultural University, and the whole experimental operation process was supervised by the Experimental Animal Ethics Committee of NEAU (NEAUEC2021 02 14). After the experiments, all animals were returned to production.

### 2.2. Animal Selection and Grouping

**Figure 1:**
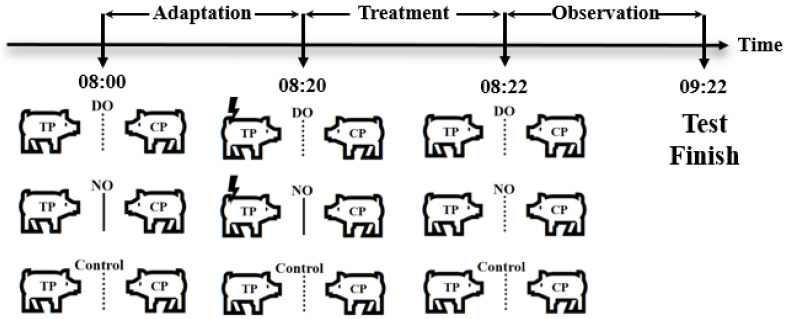
Grouping schematic, the dashed lines indicate that the pigs can only see each other, whereas the solid lines indicate that they cannot see each other. Lightning symbols represent electric shock stimulation.

Three groups were set up in the experiment, as shown in Figure 1, a direct observation group (DO group), a no-observation group (NO group), and a control group, respectively. Six pigs from each pen were randomly selected to form three paired test units, and the two pigs in each paired test unit were randomly assigned as the treated pig (TP) and the companion pig (CP), designed as two different roles (TP, CP) by two DO and NO groups, as well as the control group, for the test. In addition, the three test units were randomly assigned to the three groups on average, and a total of 27 test units from the nine pens were assigned in this way to be nine replicates in each group.

### 2.3. Test Procedure

**Figure 2:**
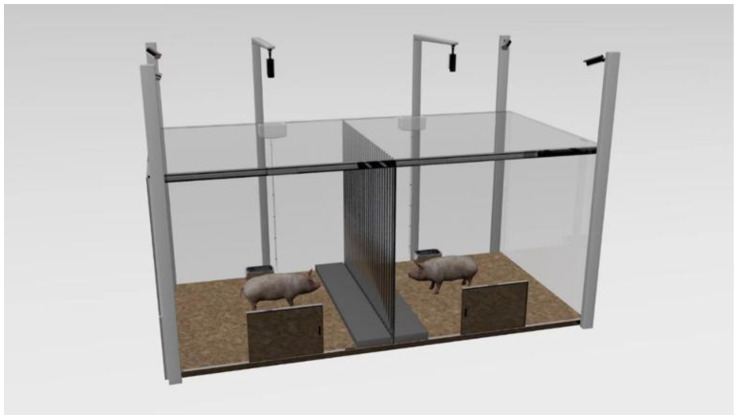
The test box, a rectangle (300 cm × 150 cm × 130 cm) made of gray acrylic boards, which is separated into two square areas by a metal bar central partition (150 cm × 130 cm, bar diameter 0.5 cm, bar spacing 5 cm). A removable acrylic board (the same size as the partition) covered with sound insulation material was fixed at the central partition in the NO group, also with the top of each square test area covered by laminated sound insulation glass to ensure that the visual and auditory isolation of the CP to the TP treatment of the NO group. A platform made of a 5 cm thick wood board (150 cm × 25 cm × 5 cm) was located on both sides of the partition, and other areas were covered with a 5 cm thick rice husk. A trough (20 cm × 17 cm × 10 cm) for ad libitum water but no feed supply and a door (40 cm × 50 cm) were set opposite to each square test area, 55 cm and 65 cm away from the partitions, respectively. In addition, six surveillance cameras at a height of 200 cm above the ground were set up at the four corners of the test box and the two tops of the two squares.

Adaption: Pigs at the 70th day of age (average weight of 26.72 ± 0.29 kg) were placed in their respective test areas for 1 h from 8:00 to 9:00 to adapt to the test box as shown in Figure 2, with no middle acrylic board and top laminated sound insulation glass setting in the test box in the adaption procedure, as well as with no feed except for the availability of water ad libitum. After that, the pigs returned to their original pens.

Treatment of different groups: The test started at 8:00 a.m. on the 71st day of age by placing two paired pigs on opposite sides of a metal partition in the test box at the same time. In the DO group, the companion pig could observe the treatment pigs suffering from the electric shock treatment, whereas the companion pig in the NO group could not observe the treatment pigs suffering from the electric shock treatment, while the two pigs in the control group were not treated and could see each other. In the DO group, there is no middle acrylic board and top laminated sound insulation glass setting in the test box during the test to ensure that the CP observed the whole process of TP subjected to electric shock treatment with no intervention by humans. After the TP and CP in each test unit adapted to the environment for 20 min, the TP was treated with electric shock (output voltage 8 V, consecutive stimulations three times, each interval 5 s, each lasting 0.2 s) for the period 8:20–8:22 a.m., whereas in the NO group, the CP was not exposed to the visual and auditory information of the treatment process to TP due to the middle acrylic board and the top laminated sound insulation glass from 8:00 to 8:22 a.m., and the TP in each test unit was treated with electric shock as in the DO group, but with no witness by the CP for the visual and auditory isolation. Then, the isolation boards were removed after the treatment in the NO group. Furthermore, pigs in the control group were left untreated from 8:00 to 8:22 a.m. After that, the behavioral observation period lasted 1 h from 8:22 to 9:22 a.m. in all three groups.

### 2.4. Behavioral Observation

Behavioral observation: Six cameras (Hangzhou Hikvision Digital Technology Co., Ltd., Hangzhou, China) in different directions recorded pigs’ behavior in the test box with continuous recording and focal sampling, and the specific behavior categories and definitions are shown in Table 1. The frequency (N) of nose–nose contact, nose–partition contact, freezing, escape attempts, and proximity, the duration (min) of simultaneous/separate/single approach to the partition, exploring and walking, and the latency (s, time from the start to the first occurrence of the action) of all the behavior were recorded.

### 2.5. Statistical Analysis

SPSS 26.0 was used for statistical analyses. All behavioral data were examined via the Shapiro–Wilk normality test and equal variance test for ANOVA, in which the frequency of escape attempts, the duration of simultaneous approach to the partition, and the latency of nose–partition contact and exploring were square-root-transformed to comply with normality. The statistical model of the two-way ANOVA was as follows: Y*_ij_* = *μ* + A*_i_* + B*_j_* + (AB)*_ij_* + *e*; Y*_ij_* is the target trait; *μ* is the overall mean; A*_i_* is the different pig roles (TP or CP, 2 levels); B*_j_* is whether the CP could witness the treatment to TP (DO or NO, 2 levels); (AB)*_ij_* is the interaction; and *e* is the random error. Furthermore, the comparison of the DO, NO, and control groups or between two different pig roles was analyzed using one-way ANOVA, and Duncan’s multiple range test was used for multiple comparisons. Furthermore, the latency of nose–nose contact, nose–partition contact, proximity, escape attempts, freezing, simultaneous approach to the partition, separate approach to the partition, and walking that could not be transformed into normality was analyzed using the Scheirer–Ray–Hare test [31]. All data were listed as the mean ± SEM. Values with *p* < 0.05 were considered significant.

## 3. Results

**Figure 3:**
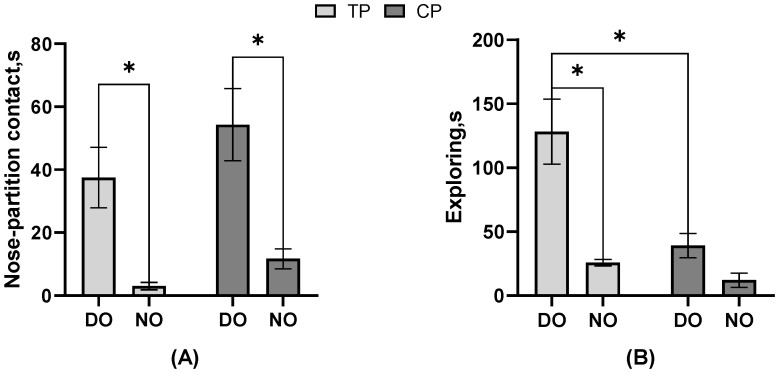
The effect of different treatments or roles on the behavioral latency of pigs. (**A**) Nose–partition contact; (**B**) Exploring; * Indicates significant differences between groups.

As the result of the behavioral frequency or duration listed in Table 2, in the DO group, nose–partition contact, separate approaches to the partition, single approaches to the partition, freezing, and escape attempts of TP were significantly more than CP, but exploring was less (all *p* < 0.05). In contrast, in the NO group, nose–nose contact, nose–partition contact, and freezing actions of TP were significantly more than CP, and the single approach to the partition was lower (all *p* < 0.05). The electric shock drove the TP to perform more nose–partition contact and freezing than CP in both DO and NO groups, unaffected by whether TP had a companion when stimulated. In TP, the nose–nose contact and separate approach to the partition of the NO group was significantly more than that of the DO group (*p* < 0.05). In CP, nose–nose contact, nose–partition contact, and walking were significantly more in the DO group than in the NO group (all *p* < 0.05), while the separate approach to the partition, single approach time to the partition, and exploring were significantly less than in the NO group (all *p* < 0.05). It can be seen that whether or not the CP observes the treatment on TP impacts the CP a lot on the behavior performance. Moreover, as shown in Figure 3, the nose–partition contact (*p* = 0.04) and exploring (*p* = 0.01) latency of TP in the DO group were significantly longer than those of TP in the NO group, and the nose–partition contact of CP in the DO group was significantly longer than that of CP in the NO group (*p* = 0.02). Both TP and CP in the NO group had a shorter period of latency for the first nose–partition contact to occur. Also, in the DO group, the exploring latency of TP was significantly higher than that of CP (*p* = 0.01).

**Figure 4:**
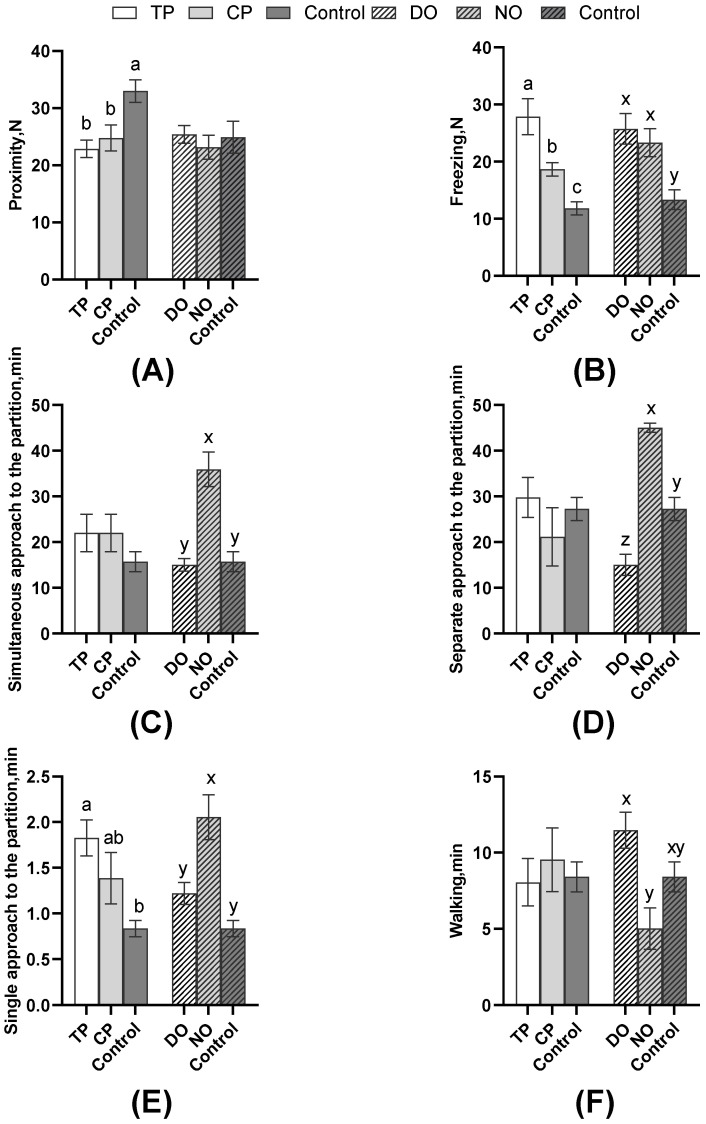
Comparison of pigs’ behavior of the DO and NO groups or different test roles with control. (**A**) Proximity; (**B**) Freezing; (**C**) Simultaneous approach to the partition; (**D**) Separate approach to the partition; (**E**) Single approach to the partition; (**F**) Walking. Different letters on the bars indicate significant differences (a/b/c: TP, CP, and control; x/y/z: DO, NO, and control).

**Figure 5:**
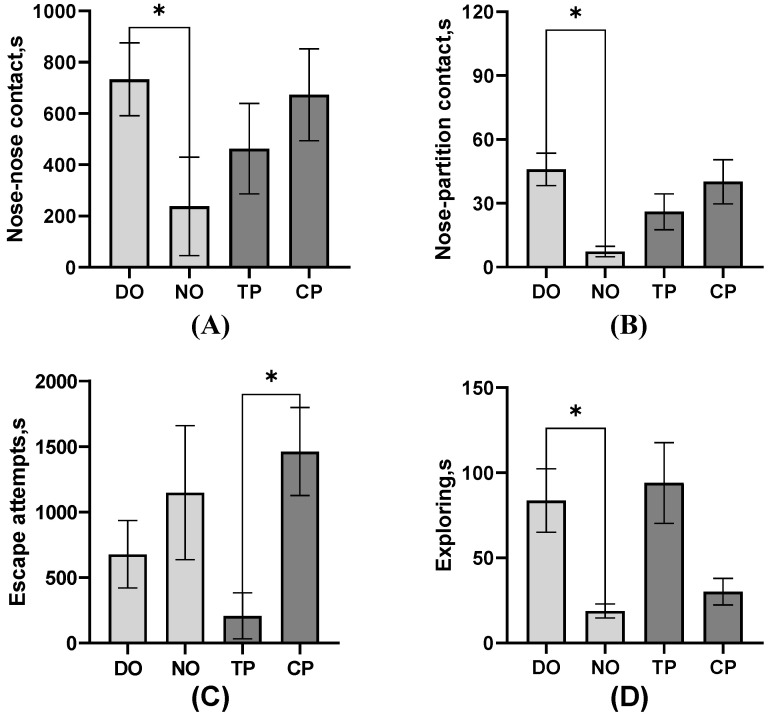
Mean-based comparison of the behavioral latency of pigs between groups. (**A**) Nose–nose contact; (**B**) Nose–partition contact; (**C**) Escape attempts; (**D**) Exploring; * Indicates significant differences between groups.

As shown in Figure 4, among the average comparison of the DO and NO groups, the freezing of the TP was significantly higher than that of the CP and control groups (*p* < 0.01), as well as the CPs (*p* = 0.01). The single approach to the partition of the TP was significantly more than the controls (*p* = 0.03), but the proximity of the TP and CP in both DO and NO groups was significantly lower than the control (*p* = 0.01). However, as shown in Table 2, nose–nose contact, nose–partition contact, and separate approach to the partition of TP were significantly more than CP (all *p* < 0.05). Combined with the results in Figure 5, TPs explored less (*p* = 0.02, Table 2) than CPs, whereas the escape attempts of TP were significantly more than CPs *(p* < 0.01, Table 2), but TPs attempted to escape significantly earlier than CPs (*p* < 0.01, Figure 5C). As for the average of the different roles in Figure 4, simultaneous, separate, and single approaches to the partition of the NO group were more than the DO and control groups. The separate approach to the partition of the control group was significantly more than the DO group (all *p* < 0.01), whereas walking was significantly less than the DO group (*p* < 0.01). Also, pigs in the DO group nosed both the companion (*p* = 0.02, Figure 5A) and the partition (*p* < 0.01, Figure 5B), and the exploring (*p* < 0.01, Figure 5D) latency was later than that in the NO group.

In addition, the frequency of nose–nose contact (*p* < 0.01) and nose–partition contact (*p* = 0.04), the duration of separate approach to the partition (*p* < 0.01), single approach to the partition *(p* < 0.01), and exploring (*p* = 0.04) were all affected by the interaction effect.

## 4. Discussion

### 4.1. The Emotional Contagion between TPs and CPs

Specific forms of inactivity expressed in response to perceived threats such as freezing appear to be, to date, the best-validated indicators of specific affective states in animals [32]. For example, one study by Jeon found that observer mice watching demonstrator mice suffering foot shocks showed a fear response of freezing [33]. Similarly, Keum et al. tested the emotional contagion of fear in mice and found that observers and demonstrators would experience a simultaneous freezing response [34]. Prairie voles in Pavlovian conditioning to fear were found to have significantly increased behaviors associated with anxiety (self-grooming) and fear (freezing), and the behavioral responses of observers were consistent with demonstrators, suggesting that similar emotional contagion was occurring between demonstrators and observers [18]. Moreover, attempts to escape from the environment can also occur when pigs are subjected to social isolation or any unpredictable aversive treatment [11,35]. These studies indicate that freezing and escape attempts are the activities under the negative emotions of pigs, and in this study, the DO group and NO group freezing were more than in the control, and TP and CP freezing were also more than in the control, indicating that regardless of the pigs’ role and whether they could see if other individuals were stimulated by the process, the pigs would show freezing to negative emotions. Furthermore, TP freezing and escape attempts occurred more often than for CPs in the experiments, suggesting that pigs directly exposed to negative treatment produce stronger fear responses. Furthermore, the control pigs attempted to escape similarly to both DO and NO groups, possibly due to incomplete adaptation to the test environment.

While latency is the time from some specified event [25], the first occurrence of freezing and escape attempts were stimulus-based responses, not emotion-based, so the shorter the latency period, the more quickly the pigs responded. The latency of escape attempts was significantly lower in TPs than in CPs, suggesting that the pigs directly receiving the punishing stimulus would respond more rapidly to the treatment. Furthermore, more freezing was seen among TPs than CPs, which suggests that the treatment pigs produced a stronger fear response than the observers; but more freezing occurred among CPs than among the control pigs, indicating that fearful emotions similar to the demonstrators may have been induced. This is similar to other studies, whereby demonstrator pigs being negatively treated by social isolation responded by standing alert (similar to freezing) and attempting to escape, and subsequently, the companion pigs, who entered after the treatment to demonstrator pigs, also performed alert standing, with companions possibly sensing the demonstrator’s negative emotions [11]. Similar to the findings of Reimert et al., Goumon et al. [4] found that when the demonstrator was seen to be restrained, the observer pigs felt distressed and showed similar behaviors to the demonstrator pigs, such as freezing and escape attempts, indicating that negative emotional contagion occurred between the demonstrator and the observer. Since emotional contagion and similar behavioral responses could occur between paired pigs, it is speculated that the CP in the test group in this study experienced emotional contagion of fear, and thus both showed a higher freezing response than the control group, and the frequencies of freezing and escape attempts were similar for the CP in the test group. More importantly, the CP in the NO group could not witness the stimulation process of the TP, and could only feel the emotion through the information contact with the TP after the treatment on TP. Thus, the fear-related behavioral performances of freezing and escape attempts were similar to those of CP in the DO group, and then the CP in the NO group, who did not observe the punishing stimulus process, also developed fearful emotions.

Fewer exploring was associated with states of restlessness or other negative emotions [36]. For example, rats in social failure explored less in new environments [37]. Similar to negative treatment, behaviors of disinterest in social interactions, behavioral inhibition, and reductions in locomotor and exploration were observed in sick animals [38]. In this study, the CPs in both DO and NO groups performed fearful emotional-related behaviors such as freezing and escape attempts. However, the CP in the DO group explored more than in the NO group, which refers to the following two possibilities: one is that the fearful emotion of the CP was not strong enough to inhibit the exploring; and the other is that, in contrast, the observational fear learning and empathy induced a very fearful emotion to enhance the pigs’ exploring latency to release stress and calm their emotions [12]. This is because it has been shown that frustration in motivation to perform specific activities tends to increase the animal’s locomotor activity while possibly reducing the time spent being inactive [32]. For instance, the research of Tripaldi [39] showed that buffalo cows spend more time in inactive idling when in intensive conditions than when they have free access to ample yards and potholes. At this point, the CP in the DO group might look like this, increasing their walking and exploring, which may have relieved their nervousness and restlessness. Similarly, the latency of TP exploring in the NO group was earlier than that of TP in the DO group, probably because of the lack of companionship when being treated, and the need to calm the distressing emotions of the TP in the NO group in the early stage was more dependent on exploring the environment.

Animals can undergo observational learning and emotional contagion without prior experience [40,41,42]. Theoretically, the CP in the DO group in this experiment may have been subject to the combined effects of the TP’s observational fear learning, emotional contagion, and cognitive empathy (cognitive empathy generated by direct observation and cognitive empathy from social interaction), whereas the behavioral responses of the CP in the NO group resulted from a combination of emotional contagion and cognitive empathy only generated from social interaction but not from direct observation without the observational fear learning. The CP in the NO group lacked cognitive empathy from direct observation compared to the CP in the DO group, and CPs in the DO and NO groups showed similar and higher escape attempts and freezing behaviors than those in the control group. From this, it is presumed that fear learning and cognitive empathy generated by observation did not mask the effects of emotional contagion and cognitive empathy generated by social interactions on the emotional responses to fear in pigs. Also, based on the previous research on empathy, the ability of emotional contagion is much more fundamental than any other cognitive contagion [4], so it is assumed that at least emotional transmission occurred between the paired pigs in the NO group.

### 4.2. Emotional Contagion and Social Support

Social support may occur when animals experience emotional contagion [15,31,43]. Nose-to-nose contact is a manifestation of social interaction, and one study found that pigs in the group with more nose–nose contact had a more significant weight gain, speculating that nose–nose contact may have a beneficial effect on pigs [44]. In Goumon’s experiment which studied the behavioral preferences of pigs, it was found that social interactions often occurred, whereby about 30% of paired units of pigs would first engage in nose–body contact, and that nose–nose contact (which did not distinguish which party initiated the experiment) occurred randomly, with the hypothesis that unidirectional snout–body interactions serve a social exploration and recognition purpose in an affiliative context [10]. The unidirectional snout–body interaction in Goumon’s study was similar to the nose–nose contact behavior here (initiated by the initiator). The partition between the two pigs limited the nose–body contact, which motivated the pigs to perform nose–nose contact, nose–partition contact, proximity, and simultaneous/separate/single approach to the partition, instead of snout–body interactions. The nose–partition contact of the TPs in the DO and NO groups was higher than that of the CPs, indicating that the TPs were eager to interact with their companions. The difference was that the nose–nose contact of TP in the NO group was higher than that of the CP, while there was no difference in the DO group, probably because the later appearance of the CP to the TP drew more attention to the TP in the NO group, and the frequent nose–nose contact was to identify the companion as well as to convey fearful emotions. At the same time, compared to the DO-group TP, this also corresponds to the increase in the NO-group TP’s separate approach to the partition. Furthermore, the social interaction behaviors of the TP in both DO and NO groups were similar except for nose–nose contact and separate approach to the partition, speculating that the companions could provide social support regardless of whether they could observe the stimulation process of the TP, and the degree of social support of the CP to the TP was similar in both DO and NO groups. However, in terms of behaviors related to social interactions between the CPs of both DO and NO groups, the companions supported the treatment pigs in different ways. The CP in the DO group had more nose–nose contact and nose–partition contact than the CP in the NO group, which indicates that direct observation was more likely to trigger fearful emotions by nose contact with the TP. Research has shown that individuals communicate to protect themselves and others from environmental influences, such as predator threats [45]. In the context of danger, individuals of social species communicate with other group members to inform them of threats using vocal, visual, and pheromone cues [46]. This may be exactly what was occurring at this time in the DO group because, as we observed the treatment process, the nose–nose contact and nose–partition contact of the CP with the TP was enhanced, and a longer latency for nose–nose contact and nose–partition contact in the DO paired unit was also seen. In contrast, the CP in the NO group was absent for the treatment process, so the duration for the separate and single approach to the partition was longer, and so was the simultaneous approach to the partition. Meanwhile, nose–nose contact and nose–partition contact latency was also shorter in the NO group of the paired units. It shows that the CPs in both DO and NO groups did not interact socially with TPs in the same way, but both generated social support for the TPs.

Thus, as mentioned above, although the observational fear learning and cognitive empathy generated through the observation of the CP in the DO group could not cover the effects of emotional contagion and cognitive empathy generated by social interactions on the behavioral responses related to the fear of freezing and escape attempts of the TP, it significantly affected social interaction behaviors such as nose–nose contact, nose–partition contact, and separate and single approaches to the partition. Moreover, both observational fear learning and cognitive empathy are cognitive-based, and this study cannot determine whether cognitive empathy exists in pigs, but at least observational fear learning of the CP in the DO group exists because pigs are capable of fairly complex cognitive processes with social learning and memory capabilities; they can learn and remember aversive events [47,48]. This leads to the speculation that observational fear learning is one of the reasons for triggering differences in the social interaction behaviors of the CPs between the DO and NO groups. Additionally, negative emotions were triggered regardless of whether the pigs were able to observe their companion suffering from a negative stimulus, but direct observation altered the frequency or duration of behaviors related to social interactions in pigs. For example, direct observation increased nose–nose contact and nose–partition contact of the CP, whereas, without the observation experience, the CP performed a more separate/single/simultaneous approach to the partition. Therefore, in pig farming, it remains to be explored whether some of the unavoidable treatment processes for the pigs should be observed by their companions is beneficial to the groups or not. And, subsequent studies will need to be validated in conjunction with physiological traits, as well as long-term experiments to explore the transmission of negative emotions in pigs with or without cognitive empathy.

## 5. Conclusions

The fear of the treated pig emerges more rapidly and is more intense than the companion for the direct punishing treatment. Whether or not the companion observed the stimulus process, it was able to undergo a similar fearful behavioral performance as the treatment pig, generate fearful emotions, and provide social support to the treatment pig. However, how social support was provided to the treated pigs varied, with companions who were able to observe the stimulus process performing more nose–nose contact and nose–partition contact to support their peers, while companions who were unable to observe the stimulus process chose to be close to their peers by the separate and single approach to the partition instead of nose contact.

## Figures and Tables

**Table 1:** Definitions of observed behaviors.

Behavior	Description
Behavior associated with social support	
Nose–nose contact (N, s)	Touching the nose of another pig with the rooting disc
Nose–partition contact (N, s)	Touching the central partition with the rooting disc
Proximity (N, s)	Head within 25 cm of center partition
Escape attempts (N, s)	Pig jumps in air or against the wall or door of a compartment
Freezing (N, s)	Standing motionless with whole body and head fixed
Simultaneous approach to the partition (min, s)	Heads of two pigs within 25 cm of the center partition simultaneously
Separate approach to the partition (min, s)	Head of one pig within 25 cm of the center partition alone
Single approach to the partition (min, s)	(Simultaneous approach to the partition + Separate approach to the partition of TP or CP)/Proximity of TP or CP
Other behavior	
Exploring (min, s)	Sniffing, nosing, or rooting the rice husk and the walls of the pen in the rice husk area
Walking (min, s)	Moving in a forward or backward direction

Note: Behavioral indicators refer to Reimert, Bolhuis, and Goumon and Spinka [4,19,25].

**Table 2:** Frequency and duration of behavior via two-way ANOVA.

		DO	NO	Mean	*p*-Value
Nose–nose contact (N)	TP	10.00 ± 1.8	24.00 ± 4.16	14.67 ± 2.86	<0.01
	CP	12.17 ± 2.3	4.33 ± 0.88	9.56 ± 2.01	0.04
	Mean	11.08 ± 1.43	14.17 ± 4.79		0.25
	*p*-value	0.47	<0.01	<0.01	
Nose–partition contact (N)	TP	25.67 ± 1.23	26.67 ± 6.01	26.00 ± 1.92	0.77
	CP	17.17 ± 1.30	7.67 ± 1.20	14.00 ± 1.83	0.01
	Mean	21.42 ± 1.54	17.17 ± 5.06		0.90
	*p*-value	<0.01	<0.01	<0.01	
Proximity (N)	TP	24.00 ± 1.59	25.67 ± 2.03	24.56 ± 1.22	0.67
	CP	28.83 ± 2.74	20.67 ± 3.48	24.78 ± 2.28	0.12
	Mean	25.42 ± 1.57	23.17 ± 2.12		0.43
	*p*-value	0.37	0.27	0.93	
Freezing (N)	TP	32.67 ± 3.12	28.33 ± 1.45	31.22 ± 2.18	0.27
	CP	18.83 ± 1.60	18.33 ± 1.76	18.67 ± 1.67	0.90
	Mean	25.75 ± 2.68	23.33 ± 2.59		0.37
	*p*-value	<0.01	0.04	<0.01	
Escape attempts (N)	TP	12.00 ± 1.61	8.00 ± 3.79	10.67 ± 1.7	0.14
	CP	3.33 ± 0.85	2.00 ± 0.58	2.89 ± 0.61	0.61
	Mean	7.67 ± 1.57	5.00 ± 2.18		0.17
	*p*-value	<0.01	0.06	<0.01	
Simultaneous approach to the partition (min)	TP&CP	15.05 ± 4.90	35.93 ± 10.37	22.01 ± 12.30	<0.01
Separate approach to the partition (min)	TP	21.52 ± 2.24	43.74 ± 1.41	28.93 ± 4.00	<0.01
	CP	8.56 ± 1.30	46.35 ± 1.11	21.16 ± 6.36	<0.01
	Mean	15.04 ± 2.31	45.05 ± 0.10		<0.01
	*p*-value	<0.01	0.43	0.02	
Single approach to the partition (min)	TP	1.56 ± 0.12	1.73 ± 0.16	1.61 ± 0.09	0.40
	CP	0.89 ± 0.09	2.38 ± 0.41	1.39 ± 0.29	<0.01
	Mean	1.22 ± 0.12	2.06 ± 0.25		<0.01
	*p*-value	<0.01	0.04	0.98	
Exploring (min)	TP	15.91 ± 1.38	14.63 ± 1.62	15.48 ± 1.03	0.76
	CP	28.17 ± 4.37	12.37 ± 3.42	22.04 ± 3.31	<0.01
	Mean	22.04 ± 2.86	13.50 ± 1.77		0.24
	*p*-value	<0.01	0.55	0.02	
Walking (min)	TP	10.36 ± 1.30	6.60 ± 1.41	9.10 ± 1.12	0.19
	CP	12.58 ± 1.99	3.44 ± 2.18	9.53 ± 2.09	<0.01
	Mean	11.47 ± 1.18	5.02 ± 1.36		<0.01
	*p*-value	0.34	0.33	0.82	

## Data Availability

The data presented in this study are available on request from the corresponding author.
